# A Monoclonal Antibody-GDNF Fusion Protein Is Not Neuroprotective and Is Associated with Proliferative Pancreatic Lesions in Parkinsonian Monkeys

**DOI:** 10.1371/journal.pone.0039036

**Published:** 2012-06-20

**Authors:** Sachiko Ohshima-Hosoyama, Heather A. Simmons, Nichole Goecks, Valerie Joers, Christine R. Swanson, Viktoriya Bondarenko, Rebecca Velotta, Kevin Brunner, Laura D. Wood, Ralph H. Hruban, Marina E. Emborg

**Affiliations:** 1 Preclinical Parkinson’s Research Program, Wisconsin National Primate Research Center, University of Wisconsin–Madison, Madison, Wisconsin, United States of America; 2 Wisconsin National Primate Research Center, University of Wisconsin–Madison, Madison, Wisconsin, United States of America; 3 Neuroscience Training Program, University of Wisconsin–Madison, Madison, Wisconsin, United States of America; 4 Department of Medical Physics, University of Wisconsin–Madison, Madison, Wisconsin, United States of America; 5 Department of Pathology, The Sol Goldman Pancreatic Cancer Research Center, The Johns Hopkins University School of Medicine, Baltimore, Maryland, United States of America; University of Nebraska Medical Center, United States of America

## Abstract

Glial cell line derived neurotrophic factor (GDNF) is a neurotrophic factor that has neuroprotective effects in animal models of Parkinson’s disease (PD) and has been proposed as a PD therapy. GDNF does not cross the blood brain barrier (BBB), and requires direct intracerebral delivery to be effective. Trojan horse technology, in which GDNF is coupled to a monoclonal antibody (mAb) against the human insulin receptor (HIR), has been proposed to allow GDNF BBB transport (ArmaGen Technologies Inc.). In this study we tested the feasibility of HIRMAb-GDNF to induce neuroprotection in parkinsonian monkeys, as well as its tolerability and safety. Adult rhesus macaques were assessed throughout the study with a clinical rating scale, a computerized fine motor skills task and general health evaluations. Following baseline measurements, the animals received a unilateral intracarotid artery MPTP injection. Seven days later the animals were evaluated, matched according to disability and blindly assigned to receive twice a week iv. treatments (vehicle, 1 or 5 mg/kg HIRmAb-GDNF) for a period of three months. HIRmAb-GDNF did not improve parkinsonian motor symptoms and induced a dose-dependent hypersensitivity reaction. Quantification of dopaminergic striatal optical density and stereological nigral cell counts did not demonstrate differences between treatment groups. Focal pancreatic acinar to ductular metaplasia (ADM) was noted in four of seven animals treated with 1 mg/kg HIRmAb-GDNF; two of four with ADM also had focal pancreatic intraepithelial neoplasia 1B (PanIN-1B) lesions. Minimal to mild, focal to multifocal, nonsuppurative myocarditis was noted in all animals in the 5 mg/kg treatment group. Our results demonstrate that HIRmAb-GDNF dosing in a monkey model of PD is not an effective neuroprotective strategy and may present serious health risks that should be considered when planning future use of the IR antibody as a carrier, or of any systemic treatment of a GDNF-containing molecule.

## Introduction

Glial cell line–derived neurotrophic factor (GDNF) is part of the transforming growth factor beta (TGFb) superfamily and has a role in the development and maintenance of mesencephalic dopaminergic neurons [1]. In animal models of Parkinson’s disease (PD), GDNF has neuroprotective and restorative properties [1], [2], [3] and is proposed as a disease-modifying strategy for PD. Because GDNF is a protein dimer with a molecular weight of 33 to 45 kDa [4] and lacks a specific carrier protein or transporter at endothelial cells, it cannot cross the blood–brain barrier (BBB). Chronic intracerebral delivery can be achieved by direct protein infusion using cannulae and pumps [2], [5] or by in vivo [6], [7], [8], [9] or ex vivo [10], [11] gene therapy methods. These approaches require invasive neurosurgical procedures, however, which is difficult to justify for early PD cases that are responsive to standard-of-care drugs [12], [13], [14].

New delivery methods for systemic GDNF dosing are being investigated. One of them is a Trojan horse technology in which the molecule of interest, in this case GDNF, is coupled to a monoclonal antibody (mAb) against a BBB cellular target that moves GDNF by transcytosis, allowing BBB transport [15], [16]. This technology was successfully tested in rodent models of PD using a chimeric monoclonal antibody against the mouse transferrin receptor fusion protein (cTfRmAb-GDNF) [17], suggesting that delivery of GDNF fusion protein may be a viable treatment option. For clinical application, the mAb against the human insulin receptor (HIR) is proposed (ArmaGen Technologies). HIRmAb is not recognized by the rodent insulin receptor, and therefore a nonhuman primate model of PD is needed for preclinical evaluation of HIRmAb-GDNF efficacy. HIRmAb-GDNF is formed by the fusion of the amino terminus of GDNF to the carboxyl terminus of the CH3 region of the heavy chain of the chimeric HIRmAb. The fusion protein is a bifunctional molecule, which binds with high affinity both to the HIR and to the GDNF receptor (15). The HIRmAb section of the fusion protein binds the BBB HIR to mediate transport to the brain, and the GDNF of the fusion protein binds to GFRalpha1 to mediate GDNF pharmacologic action (15). In the present study, we tested the feasibility of HIRmAb-GDNF to safely confer neuroprotection in a nonhuman primate model of early PD.

## Results

### GDNF Fusion Protein did not Induce Behavioral Improvements

**Figure 1:**
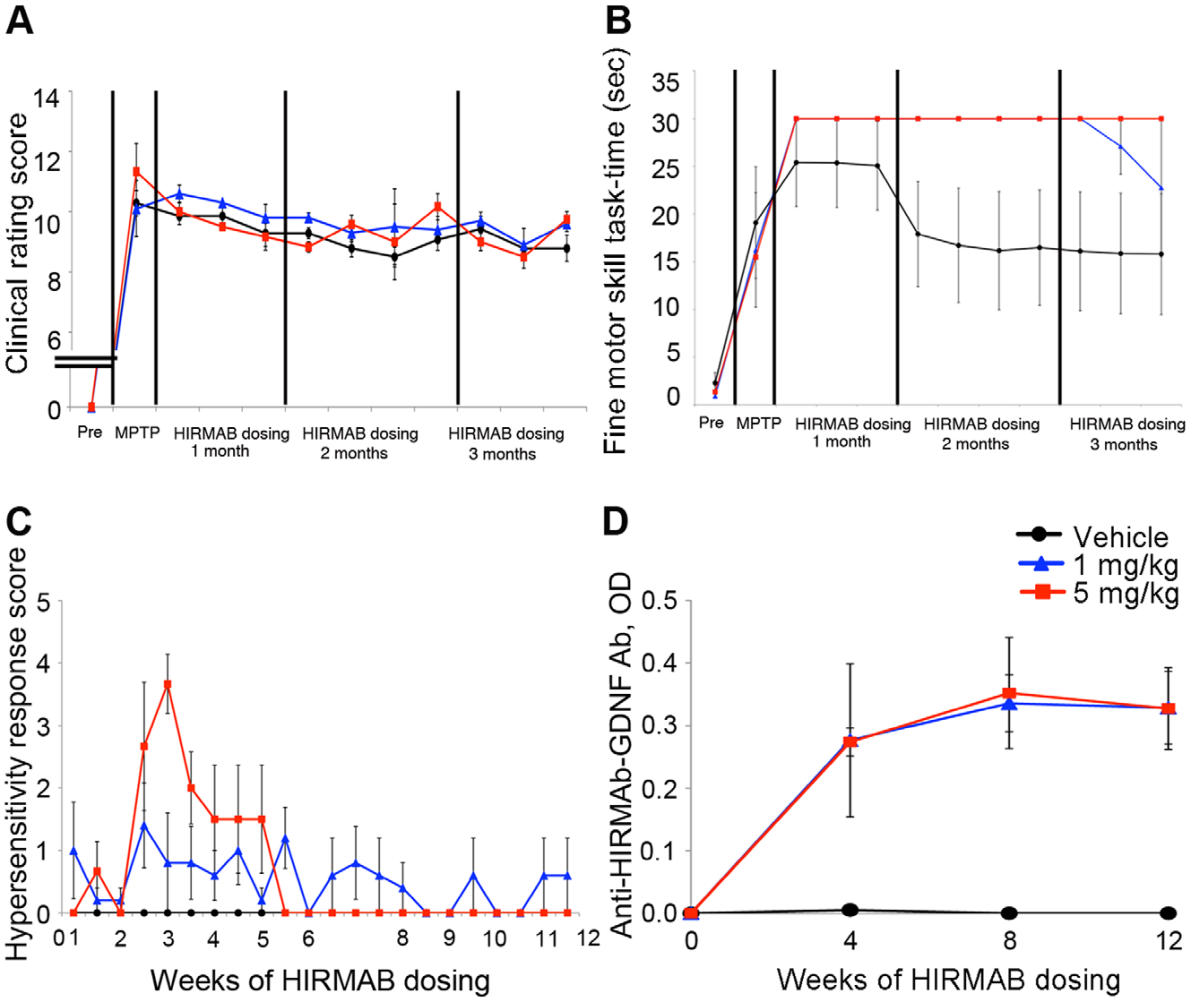
HIRmAb-GDNF does not induce improve parkinsonian signs and is associated with type I hypersensitivity reaction. (A) Clinical rating score. (B) Fine motor skills task. (C) Hypersensitivity response over time, by treatment group. (D) Optical density (OD) of HIRmAb-GDNF antibody levels in serum.

Parkinsonian signs were evaluated before and after treatment using a clinical rating scale (CRS) (Fig. 1A). At baseline, all animals presented normal behavior according to their age, scoring 0 on the CRS. At 7 days after a single intracarotid artery administration of 1-methyl-4-phenyl-1,2,3,6-tetrahydropyridine (MPTP), the monkeys showed evidence of onset of a hemiparkinsonian syndrome that included the presence of slight tremors, slowness of movement, and gait and balance disturbances. The animals that presented symptom severity corresponding to a CRS score of ≥9 points were selected, matched according to disability, and blindly assigned to a treatment group (*n*  =  7, vehicle; *n*  =  5, 1 mg/kg; *n*  =  3, 5 mg/kg) (Table 1). The CRS scores (mean±S.E) at 7 days after MPTP administration were 10.29±0.42 (vehicle group), 10.1±0.33 (1 mg/kg group), and 11.33±0.93 (5 mg/kg group). The monkeys were dosed intravenously twice a week for 11 consecutive weeks. From baseline to 8 weeks, no significant differences between vehicle and the two HIRmAb-GDNF treatment groups (*P* > 0.05, Kruskal-Wallis test) were found. At 9 and 10 weeks, no significant difference was found between vehicle and the 1 mg/kg group (*P* > 0.05, Mann-Whitney *U* test); one monkey in the 5 mg/kg group was euthanized at 9 weeks because of adverse reaction to the drug. The final scores at 11 weeks after MPTP administration were 8.79±0.43 (vehicle group), 9.6±0.29 (1 mg/kg group), and 9.75±0.25 (5 mg/kg group).

**Table 1:** Number of animals used per treatment group in the efficacy and safety experiments.

Experiment	Vehicle	HIRmAb-GDNF	
		1 mg/kg	5 mg/kg
**Treatment efficacy evaluation**			
Clinical rating scores	7	5	3
Fine motor skills	6	4	2
Morphological analysis	7	5	3
**Safety evaluation**			
Hypersensitivity response	8	7	4
GDNF and antibody levels	8	7	4

The fine motor skills of the monkeys (Fig. 1B) were evaluated using a computerized testing device. Before MPTP administration and after 2 months of training, most monkeys were able to consistently and quickly complete the task with both hands; the three exceptions were excluded from the fine motor skills analysis. The sample sizes were *n*  =  6 (vehicle), *n*  =  4 (1 mg/kg), and *n*  =  2 (5 mg/kg). As noted above one monkey in the 5 mg/kg group was euthanized at 9 weeks; because the 5 mg/kg group sample size was so small, it was excluded from the statistical analysis of the fine motor skills. Two animals in the vehicle-treated group recovered some function, especially after the second week of dosing, which in our experience is uncommon with this model [6], [8], [18], [19]. At the last fine motor skills test (week 10), average task time in seconds was, for the right hand, 0.65±0.03 (vehicle), 0.74±0.08 (1 mg/kg), and 0.45±0.00 (5 mg/kg); for the left hand, it was 15.80±6.35 (vehicle), 22.74±7.26 (1 mg/kg), and 30.00±0.00 (5 mg/kg). No significant difference was found between vehicle and the 1 mg/kg treatment group (*P*  =  0.162 ANOVA).

### A Hypersensitivity Response was Observed After Chronic HIRmAb-GDNF Treatment

The first monkeys that received the 4th dose of HIRmAb-GDNF (2^nd^ week of dosing) developed a severe dose-dependent type I hypersensitivity reaction. Episodes of skin flushes, eyelid edema, vomiting, urticaria, and in some cases respiratory distress were observed during and immediately after the infusions and required diphenhydramine and epinephrine treatment. Subsequently, it was decided to limit the number of monkeys in the high-dose group to the four animals that already received the dosing and to prophylactically administer diphenhydramine (1 to 2 mg/kg i.m.) to all animals (including the vehicle-treated group). Four animals that were excluded from behavioral and anatomical evaluations because of a score of <9 points in the CRS received treatments to assess tolerability (*n*  =  1, vehicle; *n*  =  2, 1 mg/kg; *n*  =  1, 5 mg/kg). The sample sizes for the tolerability assessment were *n*  =  8 (vehicle), *n*  =  7 (1 mg/kg), and *n*  =  4 (5 mg/kg).

Overall, 7 of the 11 HIRmAb-GDNF-treated monkeys showed a hypersensitivity response to the drug. The severity of the reaction was quantitated using a hypersensitivity response scale (see Methods section). A significant difference in the score was found at 2.5 weeks (*P*  =  0.045, Kruskal-Wallis test) and at 3 weeks (*P*  =  0.09) between vehicle and the 5 mg/kg group and at 5.5 weeks between vehicle and the 1 mg/kg group (*P*  =  0.006, Mann-Whitney *U* test) (Fig. 1C). The magnitude of the reaction decreased over time, suggesting progressive tolerability to HIRmAb-GDNF and a positive effect of the prophylactic antihistamine treatment. The exception was monkey rh2134 from the 5 mg/kg treatment group, that developed severe anaphylactic reactions necessitating discontinued dosing after dose 5. Serum ELISA against anti-HIRmAb-GDNF antibodies revealed development of circulating antibodies against the drug in all HIRmAb-GDNF-treated animals (Fig. 1D). Anti-HIRmAb-GDNF antibodies at 4 weeks and hypersensitivity response at 2 to 3 weeks were significantly correlated (Pearson’s correlation coefficient *R*  =  0.548, *P*  =  0.034).

**Figure 2:**
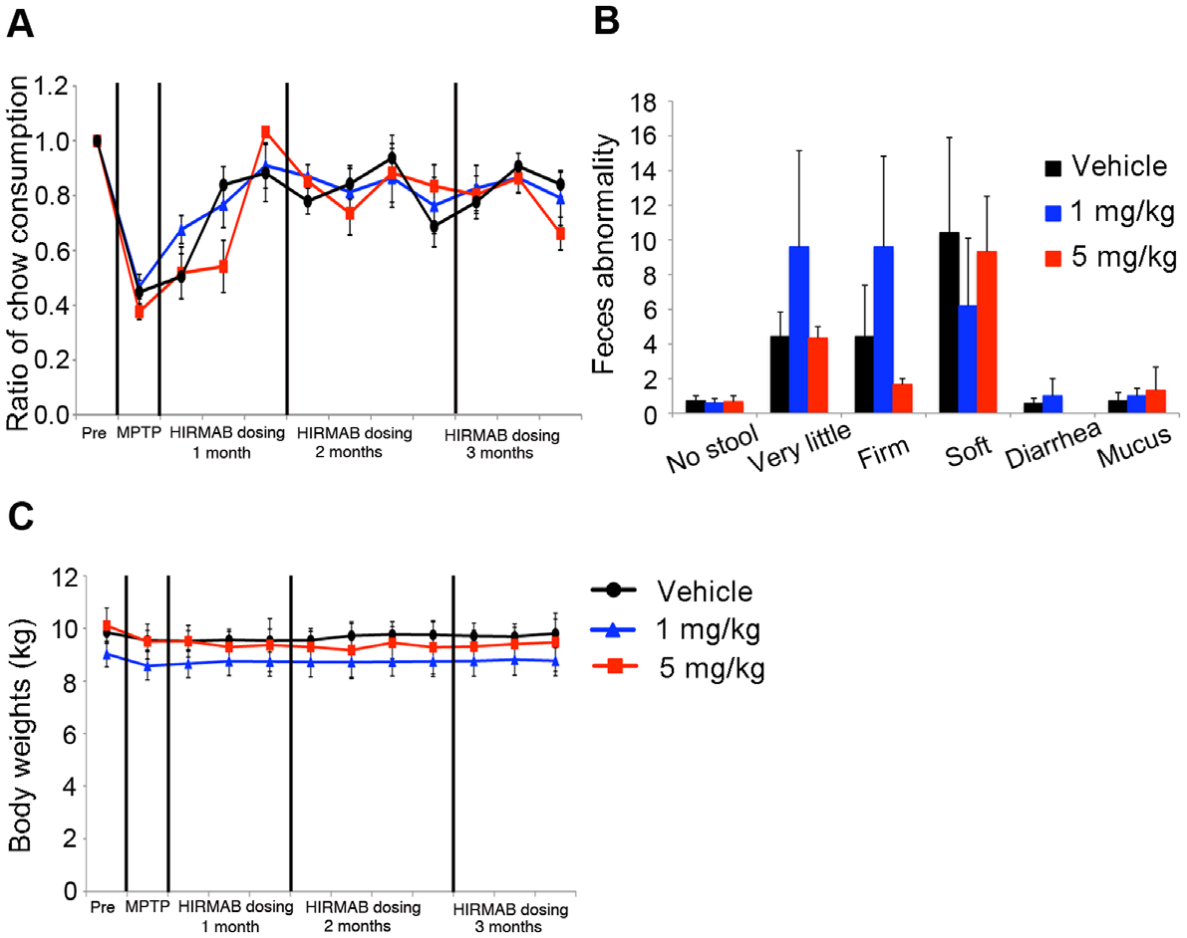
HIRmAb-GDNF treatment did not affect food consumption, feces output, or body weight. (A) Ratio of food consumption overtime (average per week after treatment/average at baseline) (B) Frequency of abnormal feces. (C) Body weight over time.

No significant differences in food intake, feces output, or weight were observed between groups (Fig. 2). Blood chemistry, glucose tolerance test, and urinalysis results were also unaffected by treatment.

### A Similar Loss of Dopaminergic Striatal and Nigral Markers was Observed Between Treatment Groups

**Figure 3:**
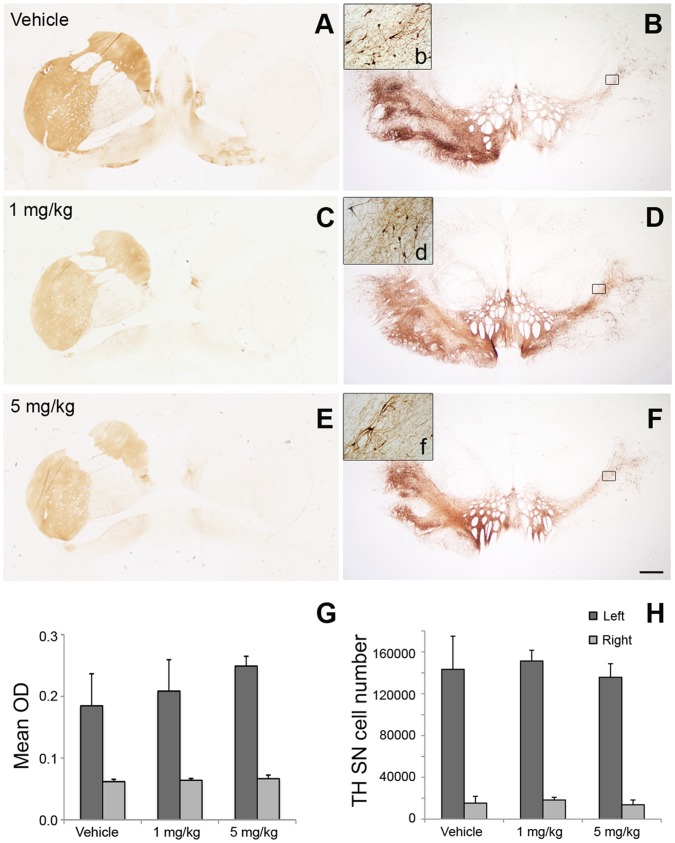
Tyrosine Hydroxylase (TH) expression is not affected by HIRmAb-GDNF. (A to F) Coronal images of the TH immunostained striatum at the level of the anterior commissure (A, C, and E) and of the substantia nigra at the level of the red nucleus (B, D, and F) of monkeys treated with vehicle (A and B), 1 mg/kg (C and D), or 5 mg/kg (E and F) HIRmAb-GDNF. Scale bar: 2.5 mm (A, C, and E); 1 mm (B, D, and F); 110 µm (insets b, d, and f). (G) Average TH optical density (OD) in the caudate and putamen nucleus. (H) Stereological cell counts of TH-positive neuron cells in the substantia nigra (SN).

**Figure 4:**
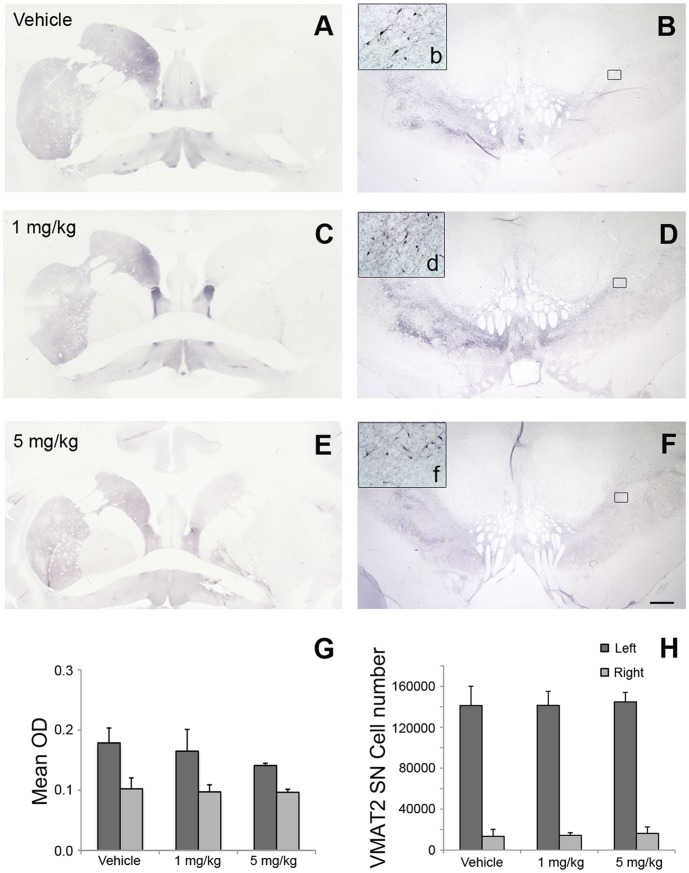
VMAT2 expression is not affected by HIRmAb-GDNF treatment. (A to F) Coronal images of the striatum at the level of the anterior commissure (A, C, and E) and of the substantia nigra at the level of the red nucleus (B, D, and F) stained with the dopaminergic marker VMAT2 of monkeys treated with vehicle (A and B), 1 mg/kg (C and D), or 5 mg/kg (E and F) HIRmAb-GDNF. Scale bar: 2.5 mm (A, C, and E); 1 mm (B, D, and F); 110 µm (insets b, d, and f). (G) VMAT2 optical density (OD) in the caudate and putamen nucleus. (H) Stereological cell counts of VMAT2-positive neuron cells in the substantia nigra.

At 3 months after initial treatment, all the monkeys were deeply anesthetized with pentobarbital (up to 35 mg/kg i.v.) and were euthanized by transcardiac perfusion with heparinized phosphate buffer solution. A full necropsy was performed; the brains were harvested and processed for morphological analysis. Dopaminergic nigrostriatal innervation was assessed with immunostaining for tyrosine hydroxylase (TH) (Fig. 3) and vesicular monoamine transporter 2 (VMAT2) (Fig. 4). In all the monkeys, qualitative observation of both markers revealed a unilateral, extensive loss of positive fibers in the caudate and putamen nucleus, as well as a loss of immunoreactive neurons in the substantia nigra.

Optical density of striatal TH and VMAT2 immunoreactivity confirmed a significant unilateral loss across groups (*P*  =  0.001, Wilcoxon signed-rank test). No significant differences were found between treatments (*P* > 0.05, ANOVA) (Fig. 3G and 4G).

Stereological cell counts of TH-positive and VMAT2-positive cells in the right side (MPTP-treated) of the substantia nigra showed significantly fewer such cells, compared with the left (intact) side (*P*  =  0.001, Wilcoxon signed-rank test). No significant difference was found between treatment groups for either marker (*P* > 0.05, ANOVA) (Fig. 3H and 4H).

### GDNF Intracerebral Levels did not Differ Between Treatment Groups

**Figure 5:**
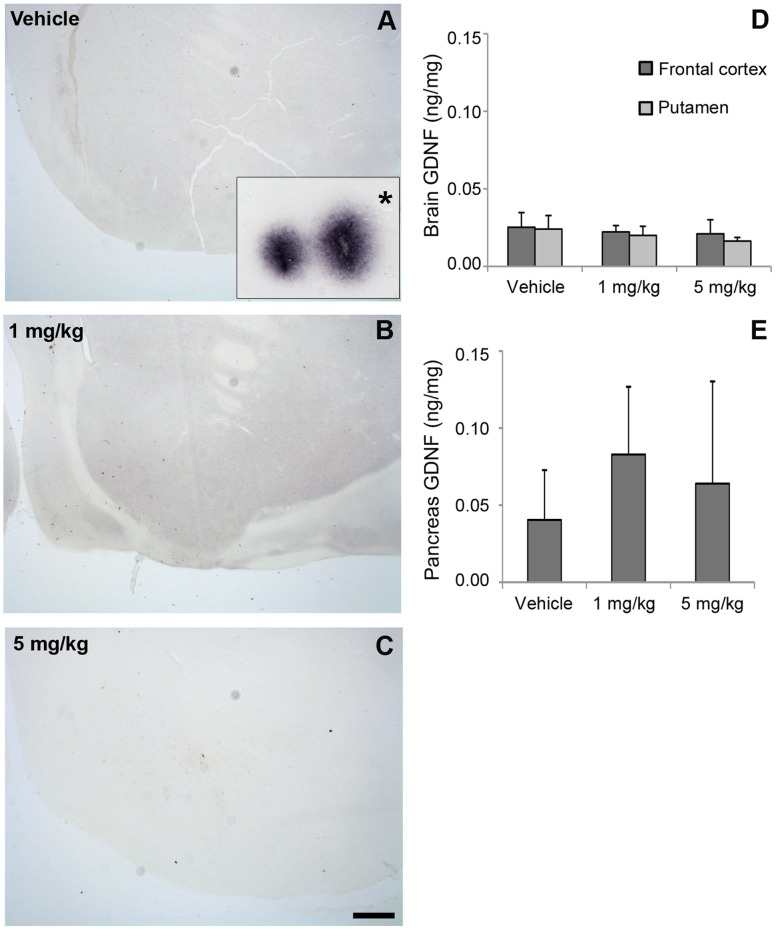
GDNF was not intracerebrally detected by immunohistochemistry or ELISA methods. (A to C) Coronal images of GDNF immunostained striatal sections of vehicle (A), 1 mg/kg (B), and 5 mg/kg (C) HIRmAb-GDNF treatments. *Inset in (A) corresponds to a positive control tissue stained in parallel from a monkey that received intracerebral injections of human neuroprogenitor cells expressing GDNF. Scale bar: 1 mm. (D and E) ELISA determination of GDNF levels in the brain (D) and in the pancreas (E).

**Figure 6:**
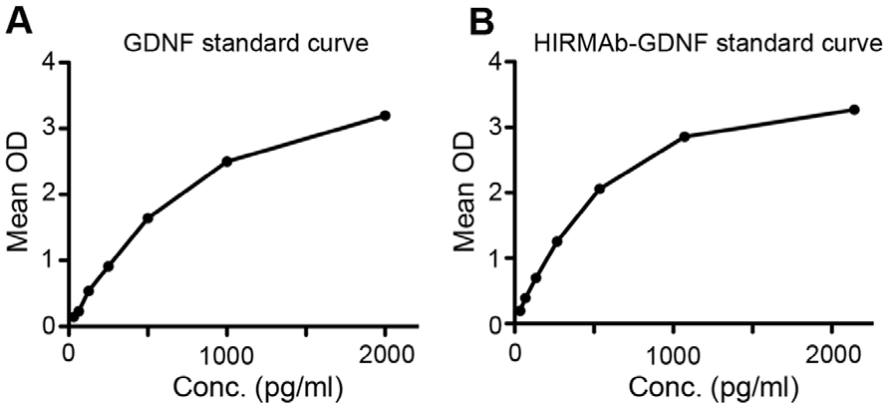
ELISA GDNF and HIRmAb-GDNF standard curves, optical density (OD) versus concentration.

Immunohistochemistry of coronal brain slices was negative for GDNF in all monkeys (Fig. 5, A to C). GDNF ELISA (Fig. 6) showed no significant difference between treatment groups (*P* > 0.05, ANOVA) (Fig. 5D) in the frontal cortex [0.025±0.009 ng/mg (vehicle), 0.022±0.004 ng/mg (1 mg/kg), and 0.021±0.00 ng/mg (5 mg/kg)] or the putamen [0.024±0.009 ng/mg (vehicle), 0.020±0.006 (1 mg/kg), and 0.01±0.002 (5 mg/kg)]. GDNF levels in the pancreas of the 1 mg/kg group showed a trend toward higher levels, compared with the vehicle group (*P*  =  0.06, ANOVA) (Fig. 5E). Serum GDNF levels were below the limit of detection in all animals.

### Several HIRmAb-GDNF-treated Monkeys Presented with Focal Metaplastic and Neoplastic Pancreatic Lesions, Myocarditis, and Hepatitis

**Figure 7:**
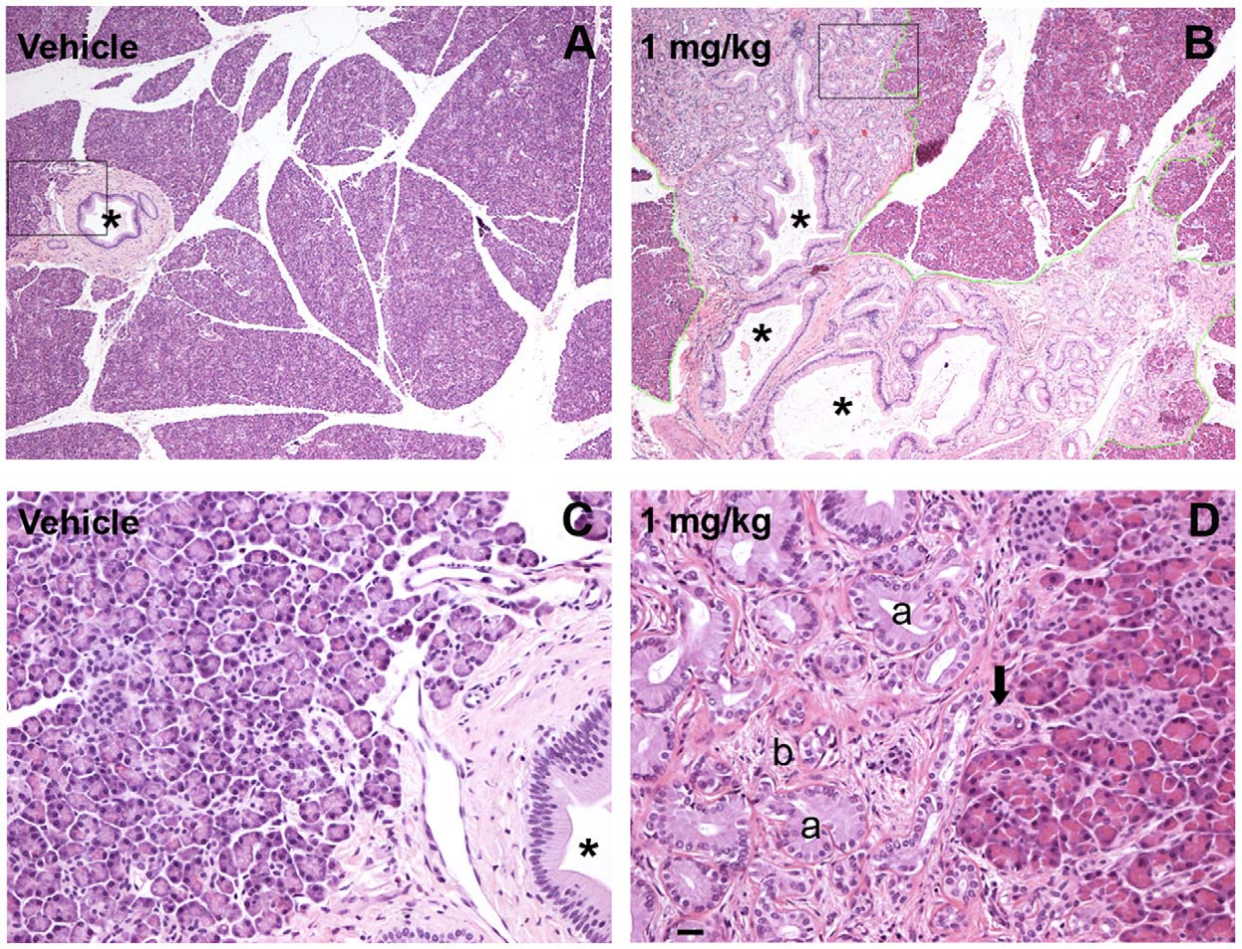
GDNF fusion protein dosing was associated with pancreatic lesions in low dose treatment groups. (A and B) Microphotographs of H&E-stained pancreas from monkeys treated with vehicle [r02048 (A) normal pancreas] or 1 mg/kg HIRmAb-GDNF [r99022 (B) pancreas with ADM]. Scale bar: 125 µm. (C normal pancreas and D pancreas with ADM) Higher-magnification views correspond to the boxed areas in (A) and (B). Scale bar: 25 µm. In the HIRmAb-GDNF-treated animal (B), note the extensive acinar to ductular metaplasia (green outline) surrounding large pancreatic ducts (*) and extending irregularly into the exocrine parenchyma; the corresponding higher-magnification image (D) shows the junction between normal pancreas (right) and acinar to ductular metaplasia (left), with a lobule of acinar cells replaced by cells with ductal differentiation (a) surrounded by fibrous stroma (b) and an exocrine acinus with both acinar and ductal cell morphology (arrow).

Gross postmortem examinations and blind histologic evaluations of major organs were performed on all animals, including the four animals that did not reach a sufficient PD score and were blindly assigned to a treatment group for additional safety assessment (Table 1). Since no gross abnormalities were noted in the pancreata, one random section from each pancreatic region (head, body, and tail) was evaluated histologically. A remarkable histologic finding was the presence of very focal pancreatic acinar to ductular metaplasia (ADM) in four of the seven monkeys in the 1 mg/kg treatment group. ADM lesions were noted in pancreatic sections of the body (n = 2), head (n = 1) and tail (n = 1). The lesions found in the pancreatic body covered an area of 40.5 mm^2^ (approximately 0.3×0.3 mm) in one case and 8,022 mm^2^ (5.6×3.2 mm) in another. The ADM lesion located in the pancreatic head had an area of 826.9 mm^2^ (1.4×0.8 mm) while the one in the tail measured 706.1 mm^2^ (1.2 mm×1 mm). These lesions were characterized by focal replacement of a lobule of exocrine acini by one of mucin-producing ductal epithelium, surrounded by fibrous stroma. There were lobules of complete replacement of acini with duct epithelium as well as acini with both acinar cells and duct cells (Fig. 7). Perhaps more importantly, intraductal papillary changes <5 mm in diameter, consistent with pancreatic intraepithelial neoplasia 1B (PanIN1B) were identified in two of these four animals. The PanIN1B lesions were present in the tissue sections where ADM lesions were identified, one in the pancreatic head and one in the body.

All other histologic alterations in the pancreata, including periductal lymphocytic inflammation and islet enlargement, were mild and distributed among animals in all treatment groups. None of the pancreatic sections in any of the animals had interstitial abnormalities, acute pancreatitis (neutrophil rich inflammation with necrosis and/or hemorrhage), or chronic pancreatitis (inflammation with irregular interstitial fibrosis and irreversible destruction of the exocrine parenchyma)^28^.

**Figure 8:**
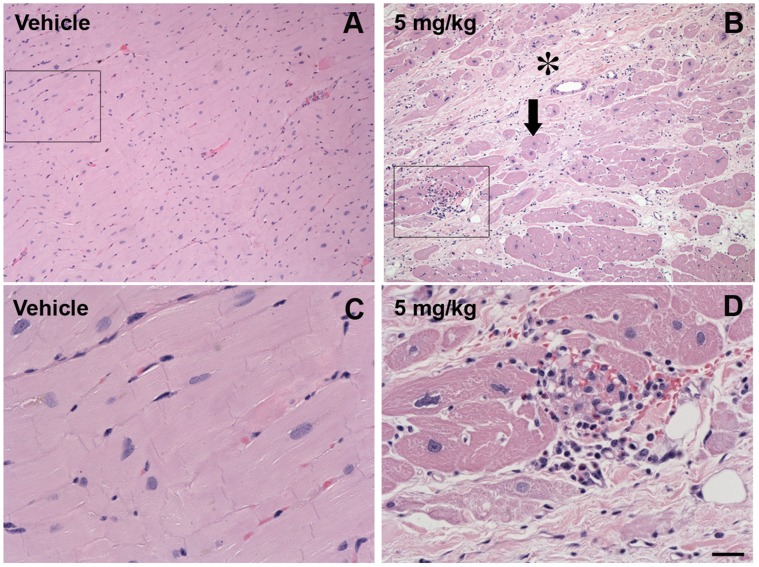
GDNF fusion protein dosing was associated with myocarditis. (A to D) Microphotographs of the heart of monkeys treated with vehicle (A and C) or 5 mg/kg HIRmAb-GDNF (B and D) (H&E staining). The HIRmAb-GDNF-treated animal [rh2134 (B)] shows a moderate multifocal degeneration with interstitial fibrosis (*) and individual myocardiocyte hypertrophy (arrow). Higher-magnification images in (C) and (D) correspond to boxed areas in (A) and (B). Note the presence of lymphocytes, plasma cells, and rare eosinophils in (D). Scale bar: 100 µm (A and B); 25 µm (C and D).

Nonsuppurative myocarditis was noted in four of the monkeys in the 5 mg/kg group and in one of the 1 mg/kg group. All of these animals had exhibited hypersensitivity responses to HIRmAb-GDNF and had lesions varying from very mild focal perivascular lymphocytic infiltration to moderate multifocal degenerative and fibrosing lymphoplasmacytic myocarditis characterized by myocardiocyte necrosis, interstitial fibrosis, individual myocardiocyte hypertrophy and rare eosinophils (Fig. 8).

**Figure 9:**
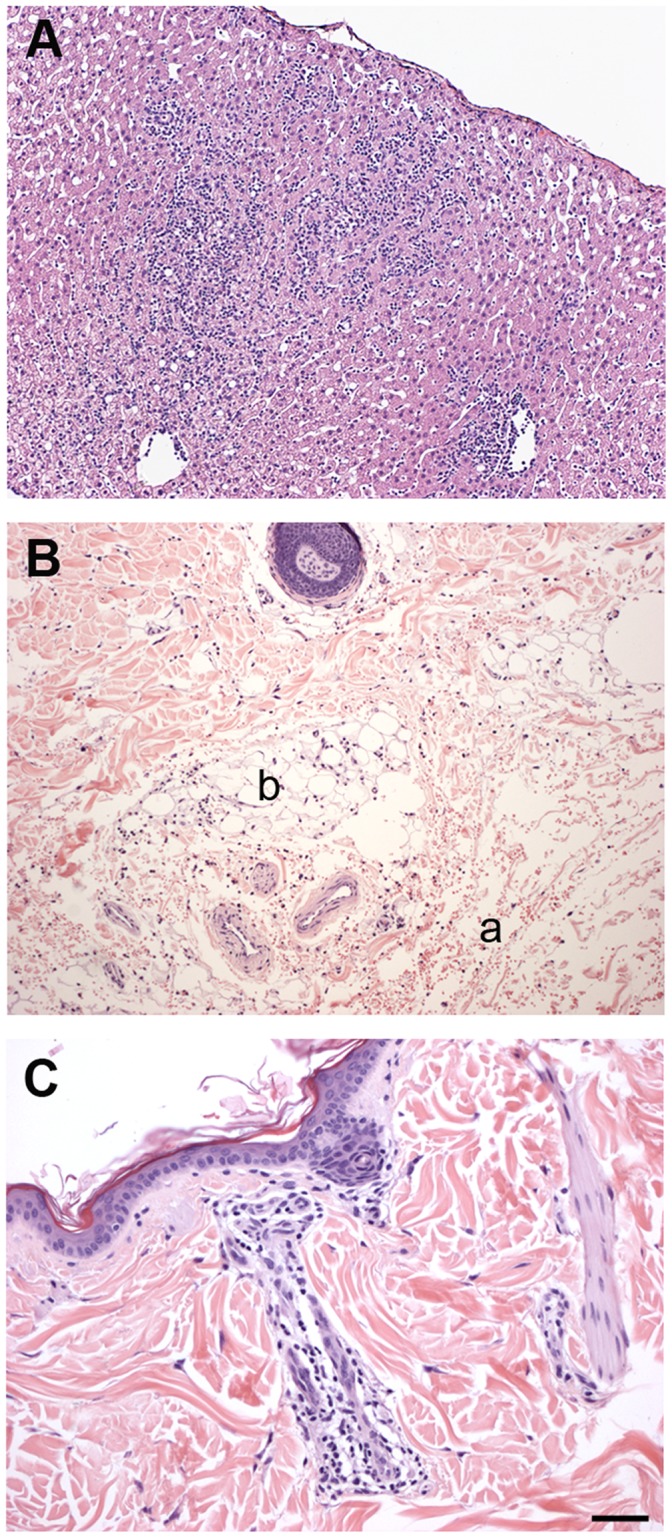
GDNF fusion protein dosing induced response lesions in the liver and the skin, associated with anaphylactic responses. (A to C) Microphotographs of the liver (A) and the skin (B and C) of a monkey (rh2134) treated with 5 mg/kg HIRmAb-GDNF (H&E staining). Scale bar: 100 µm (A and B); 50 µm (C). (A) Liver shows mild multifocal lymphocytic hepatitis with necrosis of individual hepatocytes. (B) Dermis shows subdermal hemorrhage (a) saponification of tissue adipocytes, and mild lymphocytic dermatitis. (b). (C) Dermis shows mild lymphocytic perivascular and periadnexal dermatitis.

The animal (rh2134) with the most severe anaphylactic response (had his dosing interrupted after dose 5) had, in addition to moderate myocarditis, mild multifocal lymphocytic hepatitis with individual hepatocyte necrosis (Fig. 9A) and skin lesions in the lumbar region, consistent with a type I hypersensitivity reaction, multifocal subdermal hemorrhage, and mild superficial perivascular lymphocytic dermatitis (Fig. 9, B and C). These lesions were observed at necropsy 7 weeks after cessation of HIRmAb-GDNF dosing. An additional animal (r02104) in the 1 mg/kg group had mild multifocal lymphocytic necrotizing hepatitis.

## Discussion

Our results demonstrate that administration of HIRmAb-GDNF biweekly for a period of 3 months to MPTP-treated monkeys does not confer behavioral or anatomical neuroprotection. A dose-dependent type I hypersensitivity (anaphylactic) response, associated with the development of antibodies against HIRmAb-GDNF, was observed starting during the fourth dose of HIRmAb-GDNF in all four animals in the 5 mg/kg treatment group and in four of the seven animals in the 1 mg/kg group. Focal pancreatic ADM, PanINs, myocarditis, and hepatitis were identified histologically in HIRmAb-GDNF-treated monkeys.

Intracerebral delivery of GDNF has been shown to have antiparkinsonian effects in several animal models of PD, including MPTP-treated monkeys [1], [2], [3]. Studies in rodent models with cTfRmAb-GDNF, a compound analogous to HIRmAb-GDNF, have also shown neuroprotective properties [17]. Moreover, HIRmAb-GDNF activity has been previously documented in cell culture assays and in a rat model of stroke, using direct intracerebral injection [15]. Nonetheless, no neuroprotective or antiparkinsonian effects were detected in the present study in monkeys. It should be noted that two animals in the vehicle-treated group recovered some function in the fine motor skills task, a finding that in our experience is unusual [18], [19], [20]. Morphological analysis confirmed that these two monkeys had significant dopaminergic lesions, comparable to those of other subjects. The partial functional improvement may have been facilitated by diphenhydramine hydrochloride dosing, because this drug has known antiparkinsonian properties related to its central anticholinergic activity (21,22,23).

Several possible explanations can be proposed for the lack of efficacy of HIRmAb-GDNF, including limitations of the testing paradigm, low dose of HIRmAb-GDNF, GDNF inactivation by immune reaction, or low BBB penetration of GDNF due to antibody blocking to the HIR and/or transcytosis.

The experimental design used for the present study replicates a previously validated paradigm for GDNF efficacy [6], [8]. Using this paradigm, our research group has previously shown that delivery of GDNF by in vivo gene therapy methods, starting 1 week after MPTP challenge, can prevent the functional and neuroanatomical effects of MPTP in young monkeys [6] and in aged monkeys [8]. In this paradigm, clinical effect is associated with at least 2 ng/mg (total protein) of GDNF in the putamen nucleus [6], [8]. These reports suggest that the present experimental design was appropriate and that, if effective dosing of GDNF is achieved, the treatment should be efficacious.

The dosing and the method of administration of HIRmAb-GDNF were based on previous biosafety and pharmacokinetics monkey studies that showed effective BBB penetrance without adverse effects. Administration of 0.2 mg/kg of [^125^I]-HIRmAb-GDNF led to a 10-fold increase in GDNF concentration in the brain, compared with administration of [^125^I]-GDNF [21], [22]. At 47 hours after administration of 10 mg/kg HIRmAb-GDNF, the GDNF concentration in cerebrospinal fluid ranged from 1.2 to 3.6 ng/mL; after a dosing of 0.4 or 2 mg/kg, it was below the limit of detection (1 ng/mL) [21], [22]. In the present study, the lack of detection of GDNF 24 hours after 1 mg/kg dosing could be explained if the drug was already metabolized and the levels were beyond the sensitivity of the method.

A difference between the present study and previous reports is that the brains and all organs were collected after saline perfusion, which would decrease the risk of drug retention within the vasculature and thus reduces the potential for contamination of tissue samples. This collection method may have also affected pancreatic GDNF levels; we found only a trend toward higher levels in HIRmAb-GDNF-treated animals, compared with vehicle treatment. A higher dose, such as the reported 10 mg/kg (25), might induce higher intracerebral levels of GDNF, and thus provide greater neuroprotection; however, the severity of the immune reaction in macaques to HIRmAb-GDNF at the 5 mg/kg dose precludes attempting that strategy (unless with concurrent aggressive immunomodulating therapy to prevent the development of type I hypersensitivities, especially anaphylactic reactions).

Evaluation of potential antibody formation and toxicity is accepted standard practice during preclinical testing of biologically-derived therapies, like HIRmAb-GDNF, as the chronic administration of these treatments are associated with the development of antidrug antibodies that may affect the efficacy and toxicological profile of the test article (26–28). The immunological reaction against HIRmAb-GDNF may have affected its neuroprotective properties in several possible ways. The antibodies may have bound to HIRmAb-GDNF, neutralizing the drug and/or facilitating its metabolism. If the antibodies inhibited HIRmAb capacity to bind to its BBB target, the drug would not have reached the nigrostriatal system, decreasing its efficacy. It should also be mentioned that while some of the observed adverse effects may have been facilitated by the immune response (see below), others may have been underestimated due to changes in pharmacokinetics.

Necropsy and histology revealed the presence of acute to subacute nonsuppurative myocarditis in the four animals that received 5 mg/kg treatment and in one animal that received 1 mg/kg treatment. Severe allergic or anaphylactic reactions can induce myocarditis [23], which may explain the finding in the present study. Although GDNF cardiac adverse effects have not been previously reported, systemic delivery of HIRmAb-GDNF induces levels in the heart 30 times higher than does GDNF [21], thereby increasing the potential of adverse effects. Whereas the GDNF receptor Ret is located on cardiac ganglion neurons [24], the insulin receptor (IR) is present on vascular endothelial cells and cardiac myocytes [25]. Given that muscle inflammation and high carbohydrate requirements are associated [26], it is possible that a high dose of a fusion protein that links to the insulin receptor may also be involved in the onset of myocarditis.

Pancreatic metaplasia and neoplasia appear to be rare in rhesus macaques, and neoplastic lesions are especially uncommon in macaques under 10 years of age [27]. A retrospective search of 30 years of biopsy and necropsy records (2,540 animals) at the Wisconsin National Primate Research Center (WNPRC) yielded no diagnoses of pancreatic acinar to ductular metaplasia (ADM) or pancreatic intraepithelial neoplasia 1B (PanIN1B). ADM is characterized by abnormal transformation of mature acinar cells to cells with ductal differentiation [28], similar to our histologic findings in the four monkeys treated with 1 mg/kg HIRmAb-GDNF. Although ADM can be secondary to duct obstruction and other mechanical processes, ADM has also been linked to pancreatic neoplasia. ADM and lobulocentric atrophy are frequently observed in association with PanIN lesions, and ADM is a prominent component of many genetically engineered mouse models of pancreatic cancer, where such lesions often precede the appearance of PanINs [29], [30], [31].

In contrast to ADM, PanIN lesions in humans are currently regarded as neoplasms that can be precursors to invasive pancreatic cancer [32]. The PanIN classification system, which was created to facilitate consistent diagnosis, is built upon clinical and morphological observations, as well as molecular genetic studies of duct lesions. The grading of PanINs reflects both the increasingly atypical morphology as well as the increasing prevalence of mutational events with the highest grade PanINs most closely resembling pancreatic cancer (invasive ductal adenocarcinoma) [33], [34]. Although there is a striking morphologic resemblance between the lesions in this report and PanINs in mice and humans, further studies are necessary to determine whether the pancreatic lesions observed in macaques contain the same clonal genetic mutations or other more definitive markers of true neoplasms, as noted in human pancreata with PanINs and invasive adenocarcinoma. Such genetic evidence would strengthen the morphologic impression that the pancreata of these macaques contain focal neoplastic premalignant lesions.

The occurrence of metaplastic and neoplastic pancreatic lesions in young adult rhesus monkeys chronically exposed to HIRmAb-GDNF suggests a drug-related effect. There are no previous reports of pancreatic toxicity of GDNF, but most safety studies administered GDNF intracerebrally [35] or for a shorter period of time [22]. GDNF has been reported to promote tumor cell invasion in pancreatic cancer cell lines [36], and GDNF is strongly expressed in intrapancreatic nerves [37]. High levels of the GDNF receptor RET have been observed in pancreatic cancer cells, and genetic variation in RET has been linked to variation in proliferation and invasion in pancreatic cancer cells; because its levels of expression have a negative correlation with survival rate, RET has been proposed as a prognostic marker [37], [38]. Moreover, GDNF induces perineural invasion in pancreatic cancers, and inhibition of RET signaling suppressed perineural invasion [39]. These studies provide evidence of the role of GDNF signaling in pancreatic neoplasia but at a far later stage in the neoplastic process than the changes observed in the current study. The coupling of GDNF to HIRmAb may have facilitated the accumulation of the compound in the pancreas, because numerous insulin receptors are present in the duct cells [40], [41]. This may have also contributed to overactivation of tyrosine kinase signaling pathways that are stimulated by both GDNF and insulin, affecting duct cells normal metabolic function. It can be argued that the lesions in the monkeys were facilitated by their exposure to MPTP, a dopaminergic toxin that inhibits mitochondrial complex I [42], but several lines of evidence argue against this. Although chronic exposure to toxins has been suggested as a risk factor for pancreatic cancer (e.g.: smoking), the animals in the present study were treated with a single, not chronic, central injection of MPTP, which limited peripheral effects of the neurotoxin. Furthermore, HIRmAb-GDNF dosing was started after MPTP clearance [1 week after intoxication [43]], which minimized any potential interaction between compounds and allowed the animals to recover from the surgical procedure. None of the animals were noted to have clinical symptoms of acute or chronic pancreatitis. Complete necropsies with histology are performed on every animal that dies or is euthanized at the WNPRC, and in our extensive experience with MPTP-treated monkeys, we have not observed gross or histologic pancreatic lesions. To our knowledge, there are no reports of pancreatic lesions in MPTP-treated monkeys. Additionally, none of the vehicle-treated monkeys had ADM or PanINs.

In summary, chronic administration of HIRmAb-GDNF failed to show behavioral and anatomical efficacy and was associated with severe adverse effects that preclude further studies toward clinical translation for PD or other chronic conditions. An increase in the dose of HIRmAb-GDNF or coadministration of immunomodulating therapy may increase its efficacy, but the potential for an adverse response in nonhuman primates is considerable, in light of the severity of the type I hypersensitivity (anaphylactic) reactions and the finding of pancreatic metaplasia and PanINs after a relatively short dosing period. The specific role of the HIR antibody or of GDNF in producing the immune reactions, or more importantly the pancreatic changes, are unclear from this limited dataset. It is possible that either or both responses require a bivalent ligand. In any event, the potential for these lesions would need to be considered in any future use of the IR antibody as a carrier, or of any systemic treatment of a GDNF-containing molecule. Although at this time additional toxicological studies are needed, it may be possible that short exposure to this compound for other indications (e.g. stroke) is safe.

Our findings highlight the importance of comprehensive preclinical research in nonhuman primates to assess first-in-class therapies. While this study cannot predict whether HIR-mAb GDNF will also induce an immune response in humans, accumulated evidence using biologically-derived therapies advises caution.

## Materials and Methods

### Ethics Statement

The present study was performed in strict accordance with the recommendations in the Guide for the Care and Use of Laboratory Animals of the National Institutes of Health (1996) in an AAALAC accredited facility (Wisconsin National Primate Research Center, University of Wisconsin - Madison). The experimental protocol was approved by the Institutional Animal Care and Use Committee at the University of Wisconsin -Madison (permit no. G00564). All efforts were made to minimize the number of animals used and to ameliorate any distress.

### Subjects

Adult male rhesus monkeys (*Macaca mulatta*; 5–11 years old, 6–12 kg) were used in the present study; for animal numbers in different experiments, see Table 1. Animals were housed individually on a 12-hour light/dark cycle and received food and water ad libitum. The animals’ diet was supplemented with fruit during testing sessions. General monitoring during the study included weight, food intake, feces output and condition, blood pressure, blood chemistry, intravenous glucose tolerance test, and urinalysis.

### Behavioral Evaluations

Animals were trained and blindly evaluated using positive reinforcement. Parkinsonian symptoms (tremor, posture, gait, bradykinesia, balance, upper motor skill, and defense reaction) were assessed throughout the study with a previously validated clinical rating scale (CRS) as described [44], [45]. The scale ranges from 0 to 32, with a score of 0 corresponding to normal behavior and 32 to extreme severe parkinsonian symptoms. Observation began two weeks before any experimental intervention to establish a baseline rating score.

Fine motor skills were tested 3 days per week using a monkey movement analysis panel (mMAP) in a food retrieval task as described [45]. Each test consisted of twelve total trials alternating between arms, six per side. Delaying feeding time until after the task was completed ensured animals’ compliance with the test. Data collected included the time taken for the animal to move its hand into the chamber where the fruit was located (reaction time), the time taken to pick up the fruit while the hand was in the chamber (reception time) and the total time taken to move the hand into the chamber, retrieve the fruit and bring the hand back out of the panel and into the cage (total time). The animals were trained to a consistent level of performance before MPTP dosing took place.

### Induction of Parkinsonian Syndrome

Two months after behavioral training, 22 rhesus monkeys received a single intracarotid infusion of 3 to 4 mg of MPTP-HCl (Sigma-Aldrich, lot no. 128K1549) in 20 mL saline solution delivered at a rate of 1.33 mL/min under sterile surgical conditions as described [18]. At 1 week after MPTP administration, animals were evaluated with the CRS. The 15 animals that scored ≥9 points were selected, matched according to disability, and blindly assigned to one of three treatment groups (see Table 1). Three animals were excluded from the present study because of death after MPTP administration. Four animals were excluded from the efficacy evaluations because of clinical score of <9 points, but these animals received treatments and were included in the tolerability studies.

### HIRmAb-GDNF Treatment

At 1 week after MPTP administration, the monkeys began receiving intravenous infusions of HIRmAb-GDNF (1.0 or 5.0 mg/kg) or vehicle (acetate buffered saline) twice a week, in the morning before feeding, for a total of 22 doses over an 11-week treatment period. HIRmAb-GDNF (AGT-190) was provided by ArmaGen Technologies. The compound was manufactured in a bioreactor as described 15,22. The study drug was diluted in 50 mL of normal saline (Baxter Laboratories) and was administered by intravenous infusion at a rate of 3.0 to 3.5 mL/min. The animals were sedated with ketamine (up to 10 mg/kg i.m.) for the infusion period and were closely monitored by veterinarians.

### Hypersensitivity Response Scale

The animals’ response to HIRmAb-GDNF or vehicle treatments was scored using a hypersensitivity response scale. The scale consists of five points of increasing severity of symptoms observed after dosing: 0, no clinical remarks; 1, slow recovery from drug injection; 2, skin flushes (mainly on face); 3, skin abnormalities (red bumps, white dots) and/or edema and/or vomit; 4, respiratory abnormalities (tachypnea) and skin abnormalities and/or edema and/or vomit; and 5, respiratory abnormalities and hypotension and tonic-clonic movement of limbs and skin abnormalities and/or edema and/or vomit.

### Necropsy and Preparation of Tissue

At 12 weeks after MPTP administration and 24 hours after the final treatment dose, the monkeys were necropsied, with the exception of one subject (rh2134; 5.0 mg/kg group), which received no further treatment doses after 9 weeks because of health concerns. The animals were euthanized by transcardiac perfusion with heparinized phosphate buffer solution (PBS) under pentobarbital anesthesia (up to 35 mg/kg i.v.). Before death, blood and cerebrospinal fluid samples were obtained to evaluate GDNF levels, and serum was sampled to check for the presence of GDNF antibodies. The brains were harvested and sectioned using an acrylic glass calibrated apparatus; tissue punches of the putamen and cortex were obtained, quickly frozen and kept at −80C until biochemical analysis. The rest of the brain tissue was post-fixed in 4% paraformaldehyde for 72 hours and cryoprotected by immersion in a graded (10% to 30%) sucrose/PBS solution. The tissue slabs were cut frozen (40-mm sections) on a sliding knife microtome. All sections were stored in a cryoprotectant solution before processing [46]. Coronal brain sections were used for immunohistochemical staining according to our previously published protocols [47]. The antibodies used were against tyrosine hydroxylase (TH; 1∶20,000; ImmunoStar), vesicular amine transporter 2 (VMAT2; 1∶1,000; Phoenix Pharmaceuticals), and GDNF (1∶250; R&D Systems).

Pancreata were dissected in their entirety, weighed, sectioned into three regions (head, body, and tail) and each region bisected for either biochemical or histological evaluation. For biochemical analysis the tissue was quickly frozen and kept at −80C until processing. For histology, the samples were fixed in 10% neutral buffered formalin (10% NBF), routinely processed, embedded in paraffin, sectioned into 5 µm slices, and stained with hematoxylin and eosin.

Other body tissues collected were pituitary gland, spinal cord, injection sites, stomach, duodenum, jejunum, ileum, cecum, colon, rectum, liver, gallbladder, lung, kidneys, thyroid glands, trachea, esophagus, ascending aorta, adrenal glands, axillary lymph nodes, inguinal lymph nodes, mesenteric lymph nodes, mandibular salivary glands, spleen, tongue, skeletal muscle, urinary bladder, diaphragm, testes, epididymides, seminal vesicles, prostate gland, prostatic urethra, eyes (entire globe, including optic nerve), bone, bone marrow, thymus, and heart, as well as any lesions noted during gross examination. Tissues were fixed, processed and sectioned as for pancreata. All tissues were histologically evaluated by a veterinary pathologist (HAS) blind to the treatment condition.

### Neuroanatomical Evaluations

The optical density (OD) of TH or VMAT2-immunoreactive (ir) fibers was blindly quantified within ventral, medial, and dorsal sections of both the caudate and the putamen using NIH ImageJ software version 1.44 described [8]. The total number of TH-ir and VMAT2-ir neurons in the right and left as substantia nigra (SN) was blindly calculated using unbiased stereological cell-counting methods, as described [44], [48], [49], [50].

### Pancreatic Evaluations

A representative section from the head, body, and tail of each pancreas were first blindly evaluated by a veterinary pathologist (HAS). This initial analysis was followed by blind evaluations of the sections by two M.D. pathologists, experts in pancreatic pathology (LDW & RHH). Pancreatic sections were evaluated for histologic changes such as inflammation, atrophy, dysplasia, neoplasia, and deposition of substances such as amyloid. Changes in the exocrine acini, islets, ducts, and pancreatic interstitium were described and charted for comparison among animals. The ADM lesions were measured using SPOT advanced 4.6 software in an Olympus BX41 microscope, coupled with a SPOT Insight 2MP color Mosaic digital camera.

### GDNF and HIRmAb-GDNF Antibody ELISA

GDNF levels in frontal cortex, right medial putamen, pancreas, and sera taken from monkeys at necropsy were analyzed by ELISA (R&D Systems), according to the manufacturer’s guidelines and as described [8].

HIRmAb-GDNF antibody levels were evaluated in sera using ELISA as described [22], with minor changes. A microtiter plate was precoated with HIRmAb-GDNF (250 ng/well) overnight at 4°C and was blocked with a blocking buffer (R&D Systems). The plate was incubated for 1 hour at room temperature with the sera (1∶1000 dilution) from monkeys withdrawn during the 12-week treatment study, followed by a 1∶500,000 dilution of biotinylated goat anti-monkey IgG (LifeSpan BioScience) for 1 hour and streptavidin-horseradish peroxidase (R&D Systems). Color was visualized using a peroxidase substrate system (R&D Systems). Reactivity was detected at a wavelength of 450 nm with a reference at 570 nm.

### Statistical Analysis

All data were collected and analyzed by investigators blind to the treatment groups. Statistical significance was set at *P*<0.05. All statistical analyses were performed using PASW Statistics 18.0 software (SPSS).
